# Physiological heterogeneities in microbial populations and implications for physical stress tolerance

**DOI:** 10.1186/1475-2859-11-94

**Published:** 2012-07-16

**Authors:** Magnus Carlquist, Rita Lencastre Fernandes, Søren Helmark, Anna-Lena Heins, Luisa Lundin, Søren J Sørensen, Krist V Gernaey, Anna Eliasson Lantz

**Affiliations:** 1 Center for Microbial Biotechnology, Department of Systems Biology, Technical University of Denmark, DK-2800, Kgs. Lyngby, Denmark; 2 Division of Applied Microbiology, Department of Chemistry, Lund University, SE-22100, Lund, Sweden; 3 Center for Process Engineering and Technology, Department of Chemical and Biochemical Engineering, Technical University of Denmark, DK-2800, Kgs. Lyngby, Denmark; 4 Fermenco ApS, Kgs. Lyngby, DK-2800, Kgs. Lyngby, Denmark; 5 Molecular Microbial Ecology Group, Department of Biology, University of Copenhagen, Sølvgade 83H, DK-1370K, Copenhagen, Denmark

**Keywords:** Population heterogeneity, Cell fitness, Cell membrane robustness, Flow cytometry, Budding yeast, Reporter strain, Cell factory optimisation

## Abstract

**Background:**

Traditionally average values of the whole population are considered when analysing microbial cell cultivations. However, a typical microbial population in a bioreactor is heterogeneous in most phenotypes measurable at a single-cell level. There are indications that such heterogeneity may be unfavourable on the one hand (reduces yields and productivities), but also beneficial on the other hand (facilitates quick adaptation to new conditions - i.e. increases the robustness of the fermentation process). Understanding and control of microbial population heterogeneity is thus of major importance for improving microbial cell factory processes.

**Results:**

In this work, a dual reporter system was developed and applied to map growth and cell fitness heterogeneities within budding yeast populations during aerobic cultivation in well-mixed bioreactors. The reporter strain, which was based on the expression of green fluorescent protein (GFP) under the control of the ribosomal protein RPL22a promoter, made it possible to distinguish cell growth phases by the level of fluorescence intensity. Furthermore, by exploiting the strong correlation of intracellular GFP level and cell membrane integrity it was possible to distinguish subpopulations with high and low cell membrane robustness and hence ability to withstand freeze-thaw stress. A strong inverse correlation between growth and cell membrane robustness was observed, which further supports the hypothesis that cellular resources are limited and need to be distributed as a trade-off between two functions: growth and robustness. In addition, the trade-off was shown to vary within the population, and the occurrence of two distinct subpopulations shifting between these two antagonistic modes of cell operation could be distinguished.

**Conclusions:**

The reporter strain enabled mapping of population heterogeneities in growth and cell membrane robustness towards freeze-thaw stress at different phases of cell cultivation. The described reporter system is a valuable tool for understanding the effect of environmental conditions on population heterogeneity of microbial cells and thereby to understand cell responses during industrial process-like conditions. It may be applied to identify more robust subpopulations, and for developing novel strategies for strain improvement and process design for more effective bioprocessing.

## Background

Traditionally, a microbial population has been considered homogeneous in optimisation studies of fermentation processes. Bioprocess measurements are typically obtained as an “average” of the whole cell population, thereby neglecting the effects and phenomena at the individual cell level. However, research has shown that a typical microbial population in a bioreactor is heterogeneous in most phenotypes measurable at a single-cell level [1-3]. There are indications that such heterogeneity may be unfavourable on the one hand (reduces yields and productivities), but also beneficial on the other hand (facilitates quick adaptation to new conditions - i.e. increases the robustness of the fermentation process) [4,5]. Therefore, understanding and control of microbial population heterogeneity is of major importance for improving biological production processes, and this has led to an increased interest from industry for methods to monitor population heterogeneity [6,7].

Phenotypic heterogeneity occurs even if the micro-environment surrounding the cells is constant, and it is driven by factors such as differences in cell cycle and cell ageing. Furthermore, stochastic gene transcription, translation and post-translational modifications have an impact [2]. In industrial scale fermentation processes, phenotypic heterogeneity is further amplified as a result of deficient mixing, which leads to zones with diverse environmental conditions [8]. The microbial cells, thus, experience sudden changes in the environmental conditions as they circulate from one zone to the other. These changes may pose different types of stress (e.g. oxidative, temperature, pH) on the cells and affect their metabolism and fitness [4,8,9]. The heterogeneous environment in large-scale fermenters may lead to repeated cycles of production/re-assimilation of overflow metabolites and repeated induction/relaxation of stress responses resulting in reduced biomass yield and productivity [4,10].

Stress tolerance has previously been shown to differ depending on the physiological state of the cell, i.e. which growth phase the cells were in prior to exposure to the stress factor. For example, yeast cells in respiratory ethanol growth phase have been found to be more tolerant to freeze-thaw stress than cells in respiro-fermentative glucose growth phase during aerobic batch cultivation [11]. This may be due to differences in cell membrane robustness, which is a key phenotypic trait that determines how well the cell can cope with physical stresses (such as heat, mechanical, osmotic or freezing) [12]. Many cellular stress responses are unique for the specific stress, however, there is also a global induction of cell responses leading to cross-tolerance towards non-related stresses; a phenomenon known as the environmental stress response (ESR) [13]. In a recent study by [Zakrezewska *et al.*14], tolerance to different stresses has been shown to be inversely correlated to cell growth rate, i.e. cells growing at a slow rate display a higher resistance to a number of stresses (e.g. heat, acid, oxidative) irrespectively of the cause for the reduced growth rate. The inverse correlation to growth rate was speculated to be related to the fact that the pool of cellular resources (energy, material) is limited and needs to be distributed as a trade-off between the two cellular functions growth and survival. In a population, the individuals differ in physiological state and are therefore equipped differently to cope with subsequent exposure to harsh conditions [15] that may occur in large-scale fermentation processes or in succeeding bioprocessing operations.

The aim of the present work was to map physiological state and cell robustness distributions within a microbial population, using budding yeast *Saccharomyces cerevisiae* as model organism. *S. cerevisiae* has an outstanding importance in industrial bioprocesses. It has been used in baking and alcoholic brewing for centuries, but is also used in a wide range of newer biotechnology production applications, such as production of heterologous proteins (e.g. insulin and different vaccines) and commodity chemicals. Most of the pharmaceutical proteins produced by microbial eukaryotic cells so far approved by the FDA or EMEA are almost exclusively based on production by *S. cerevisiae*[16].

To be able to shed light into whether population heterogeneity could be a consequence of the trade-off in cell economy for growth and robustness, a dual reporter system was developed that allowed for studying the prevalence of subpopulations which are differently prepared for changes in environmental conditions. The dual reporter system was based on:

(A) the expression of green fluorescent protein (GFP) [17,18] under control of the ribosomal protein promoter RPL22A, thereby making cellular GFP level proportional to growth;

(B) loss of GFP signal in cells with permeabilised plasma membranes after exposure to physical stress.

The dual nature of the reporter strain was validated by staining with propidium iodide (PI), which clearly demonstrated that cells with permeabilised plasma membrane lost GFP signal and were PI positive, while cells with remained level of GFP were PI negative.

As a case study, the relationship between physiological heterogeneity of a microbial population and the prerequisite of the population to tolerate subsequent freeze-thaw stress (as a model of physical stress) was characterised on a single cell level. It was found that subpopulations with different level of cell membrane robustness and tolerance towards physical stress co-existed in a population, and that the distribution was changing dynamically between different phases of cultivation. The results have implications for biological processing where intact cell membranes are desirable, for example in pharmaceutical protein production. In addition, the presented methodology more generally provides an additional dimension in optimisation of microbial cell factories.

## Results and discussion

### Population dynamics during batch cultivation

In order to capture the dynamic growth responses in a population during the different growth phases of batch cultivation, a reporter strain based on the expression of GFP under the control of the promoter for the ribosomal protein gene RPL22a was used. The RPL22a promoter was chosen based on that ribosomal protein gene transcription has previously been found to be linearly correlated to growth rate, as determined by transcriptome analysis of cells grown in continuous cultivation mode under different limiting conditions [19-21]. Furthermore, ribosomal protein synthesis is believed to be regulated at the transcription level [22], which makes promoters for ribosomal proteins the ideal choice for construction of growth reporter strains.

**Figure 1:**
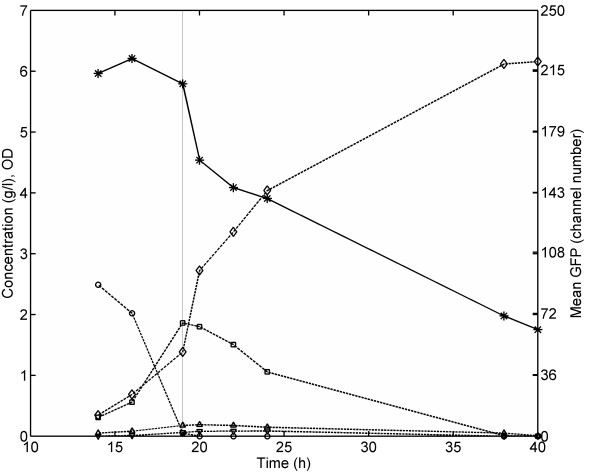
**Mean fluorescence, biomass formation, substrate utilisation and product formation during aerobic batch cultivation of the*S. cerevisiae*reporter strain in defined mineral media.** Symbols: Mean GFP fluorescence (*snowflakes*); OD_600_ (*open diamonds*); Glucose (*open circles*); Acetate (*open triangle pointing upwards*); Ethanol (*open squares*); Glycerol (*open triangles pointing downwards*). *The vertical line marks the time point glucose depletion was observed.*

To investigate the behaviour of the growth reporter strain during different growth phases, batch cultivations in well-controlled stirred tank reactors were performed and the physiology was monitored both on the whole population by standard methods and on a single-cell level by flow cytometry (see Materials and Methods). The reporter strain exhibited expected growth behaviour in defined mineral media, i.e. four distinct growth phases were observed. Growth first occurred on glucose, which was exhausted after 19 hours (Figure 1). Cells then underwent a diauxic shift and growth occurred on ethanol until stationary phase was reached approximately after 35–40 hours. The growth rate and biomass yield of the reporter strain did not differ from the control strain that did not express GFP, hence demonstrating that expression of GFP was not a burden to the cell (data not shown).

The mean GFP level defined as mean cellular fluorescence intensity measured by flow cytometry was initially quite stable around ca. 215 channel number units during growth on glucose (Figure 1). As the cells entered diauxic shift, the fluorescence decreased with approximately 30 channel number units in 2 hours. The turn-over of intracellular GFP is a result of the sum of gene transcription, gene translation as well as mRNA transcript degradation and protein degradation. Thus, the relatively rapid decrease in GFP level upon glucose depletion which coincides with a momentarily detainment of cell proliferation rate during the diauxic shift, demonstrated that the RPL22a promoter activity and GFP synthesis were rapidly down-regulated during this growth phase. The half-life of the GFP version used in the present study has previously been reported to be approximately 7 hours [23], which is coherent with the fluorescence decrease observed upon glucose depletion (approximately 15% decrease in 2 hours, Figure 1). Besides protein degradation, the decrease in GFP level may also to some extent be ascribed to dilution by cell division [24]. After the diauxic shift, the mean fluorescence continued to decrease, although at a lower rate than upon glucose depletion, which indicates that the RPL22a promoter activity and GFP synthesis were re-initiated to some extent during exponential growth on ethanol until the carbon source was depleted and stationary phase was reached. The mean fluorescence in stationary phase (ca. 70 channel number units) remained ca 10 times higher for the reporter strain compared to the auto fluorescence of the control strain.

The differences in GFP level at different growth phases confirmed that the growth reporter could be used to distinguish cells in different propagation modes, i.e. distinguish cells growing on glucose from cells growing on ethanol or in stationary phase. However, whether the lowered GFP level during growth on ethanol is a result of the lower growth rate and that the regulation of the RPL22a promoter is a part of the ‘universal’ growth rate response (GRR) as defined by[ Slavov and Botstein (2011) 25], or if the regulation of RPL22a is specific for the carbon source or other extrinsic factors is not clear. In the study performed by Slavov and Botstein (2011), RPL22a was found *not* to be a part of the ‘universal’ GRR, i.e. the transcription of RPL22a was strongly up-regulated at higher growth rates on glucose and slightly down-regulated at higher growth rates on ethanol [26]. It is therefore likely that the decreased level of GFP after the diauxic shift (Figure 1) is not strictly due to the lower growth rate per se, but also a response to the change in carbon source and the change from respiro-fermentative to respiratory metabolism. Another support for the interpretation that the regulation of the RPL22a promoter is not exclusively related to the growth rate is the observation that the GFP level decreased steadily during growth on ethanol despite the fact that the growth rate did not decrease until late exponential phase. On the other hand, the gradual decrease in GFP level during ethanol growth may additionally be due to dilution by cell division [24]. Albeit that other aspects might have influence on the regulation of the RPL22a promoter in addition to the growth rate, the reporter strain can be used to distinguish cells at different growth conditions (e.g. growth on glucose, growth on ethanol, no growth) which may be useful during optimisation of large-scale bioprocess conditions. Effects due to different stirring and feeding can be evaluated by following the response by the reporter system thereby used to guide process development. The reporter system may enable identification of cell growth physiology at single-cell level from different zones of a large-scale reactor, for example if cells are being trapped in ethanol (or alternatively glucose) rich zones for longer time periods.

**Figure 2:**
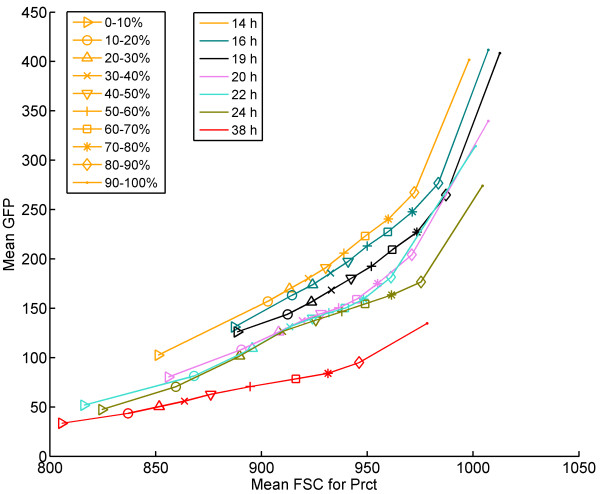
**Mean GFP level as a function of mean FSC for each percentile during aerobic batch cultivation.** For each time point there is a line (*in a different colour*) with markers for each percentile. Details of the percentile analysis can be found in the methods section and the Additional file 1: Figure S1-2.

To enumerate whether cell to cell variations in RPL22a promoter activity could be ascribed to more than differences in cell size, a percentile analysis similar to the method previously reported by [Sumner *et al.*27] was performed. For such an analysis, the events measured in the flow cytometry for each sample were first categorised in 10%-percentiles based on the forward scatter (FSC) signal; then for each percentile the mean FSC and GFP level were calculated and plotted against each other (Figure 2; see Additional file 1: Figure S1-2 for further information on how the percentile analysis was made). In Figure 2, the different coloured lines correspond to the sample time points, and each marker indicates the mean FSC and GFP for cells belonging to a given FSC percentile interval (e.g. the mean FSC and GFP of the cells presenting a FSC between the 10^th^ and 20^th^ percentile, for each time point, is indicated with an open circle). From the percentile analysis, a clear correlation between GFP level and FSC was observed for all time points, with larger cells in general having a higher GFP level (Figure 2). However, cell size was not the only determinant of GFP level as there was a discrepancy from the linearity, especially at higher FSC percentiles. The difference in GFP level that cannot be explained by cell size alone may be an artefact from the flow cytometry analysis (particularly at the 90-100% percentile), meaning that the deviation may simply be explained by the presence of cell aggregates, which are read as a single event thereby giving a false reading of the GFP level. On the other hand, it may also well indicate *non*-stochasticity and the presence of subpopulations with different regulation of RPL22a promoter activity. In relation to this, it could be speculated that larger cells with higher fluorescence propagate at a higher rate, at least during glucose assimilation, which would be in agreement with earlier observations that RPL22a transcription in glucose-limited chemostats correlated to growth rate [19,25]. Moreover, differences in cell cycle phase may contribute to the *non*-linear GFP level-cell size correlation (in general high FSC percentiles may contain a higher fraction of budding cells, which are larger than non-budding cells). In this work the budding index (BI), i.e. the fraction of budding cells in the population, was not measured, however it has been reported in the literature that for cultures in exponential growth on glucose the BI is approximately 70-80% [28,29]. This implies that budding cells would most likely be present also in the FSC percentile intervals as low as 20–30 or 30-40% and that influence by cell cycle therefore is not likely to have a large influence on the correlation discrepancies. The results reported here are however not conclusive, and additional analysis is needed to clearly state whether two cells of approximately the same FSC would present different GFP level due to the fact that one is budding and the other is not. 

Another circumstance highlighted from the percentile analysis was that the slope of the GFP level-cell size correlation differed depending on growth phase, with a lower slope during ethanol assimilation than before the diauxic shift (Figure 2). At the same time, a trend towards smaller cells throughout the cultivation could be observed. The decreased GFP level-cell size slope illustrates a decrease in growth heterogeneity, which may be a consequence of the fact that the whole population relocates resources to get prepared for survival rather than propagation. In general, the GFP level decreased to a relatively higher extent in larger cells than smaller cells, which may be interpreted as the larger cells underwent a larger physiological change (both growth and cell size decreased) as a response to the shift in environmental conditions.

### Cell to cell variation in cell membrane robustness

As a case study for determining heterogeneities in cell membrane robustness and cellular capacity to withstand physical stress, the population was exposed to freeze-thaw stress and the degree of cell membrane permeabilisation was quantified by flow cytometry after cell staining with propidium iodide (PI). It has previously been shown that there is a connection between tolerance to freeze-thaw stress and other physical stresses (H_2_O_2_ oxidative stress; calcofluor white as cell wall-challenging reagent) [30]. Therefore, cells displaying high tolerance to freeze-thaw stress are also likely to withstand other seemingly unrelated types of specific stresses since the response to most stress factors relies at least partly on the ESR [13]. Thus, mapping of subpopulations with elevated capacity to freeze-thaw stress is of high relevance for reaching more robust bioprocesses where multiple factors pose pressure on cell membrane integrity.

**Figure 3:**
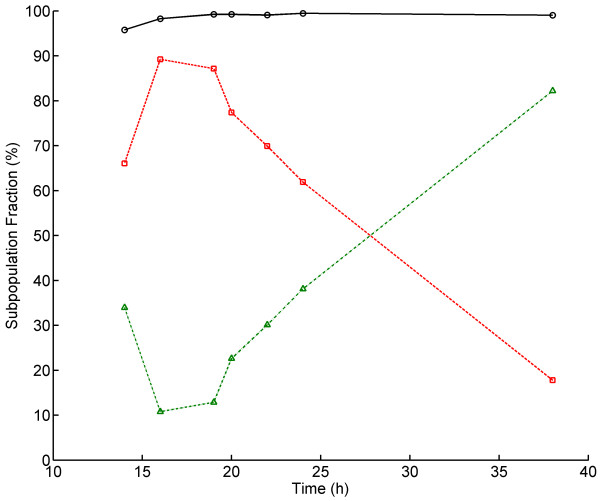
**Distribution of PI-stained cells prior to and after freeze-thaw stress exposure plotted against point of harvest during aerobic batch cultivation.** Symbols: PI negative cells prior to freeze-thaw stress (*circles*), PI negative cells (*triangles*) and PI positive cells (*squares*) after freeze-thaw stress.

**Figure 4:**
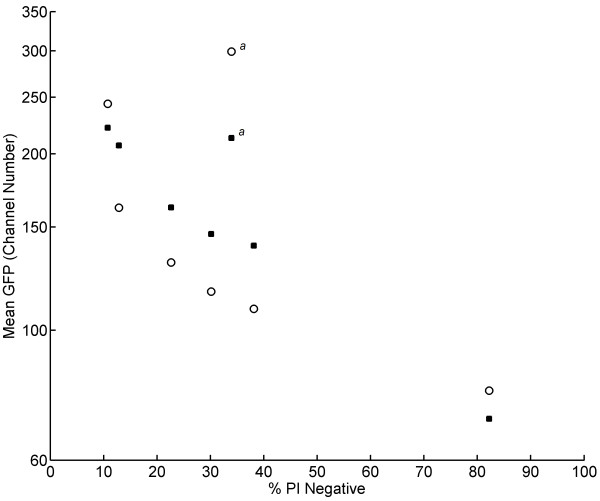
**Inverse correlation between growth and cell membrane robustness.** Mean GFP level of the whole population prior to freeze-thaw stress (*empty symbols*) as a function of the percentage of PI negative cells after freeze-thaw stress; and the mean GFP of the PI negative cells after freeze-thaw stress (*full symbols*) as a function of the percentage of PI negative cells after freeze-thaw stress. ^*a*^ These outlying data points are from the first sample of aerobic batch cultivation.

Prior to the freeze-thaw stress, over 95% of the cells were PI negative demonstrating an undamaged cell membrane phenotype (Figure 3). However, after freeze-thaw stress a significant amount of cells had permeabilised cell membranes and were thus stained with PI. The degree of cell membrane permeabilisation varied within the population and could be divided in two categories: PI negative (PI fluorescence intensity <=10), and PI positive (PI fluorescence intensity >10). Cell membrane robustness was clearly linked to the specific growth phase the cells were in prior to applying freeze-thaw stress; in general cells growing on glucose were more sensitive than cells growing on ethanol, and cells in stationary phase. The fraction of PI negative cells increased from below 20% during the exponential glucose growth phase to around 80% in stationary phase and a corresponding decrease of the PI positive subpopulation in the same time interval was seen. This is in accordance with a previously published study made on the whole population level, where viability after freeze-thaw stress was measured on cells in different growth phases [11]. Furthermore, it is in line with the hypothesis that there is an inverse correlation between growth and robustness as has been previously suggested [25]. The trade-off between growth and robustness can be further substantiated by the inverse correlation between the RPL22a promoter activity prior to stress and freeze-thaw stress tolerance (Figure 4). However, the rapid increase in cell membrane robustness after the diauxic shift may also be a consequence of the ESR, which previously has been shown to be activated during diauxia and increase tolerance to multiple stresses [13]. 

**Figure 5:**
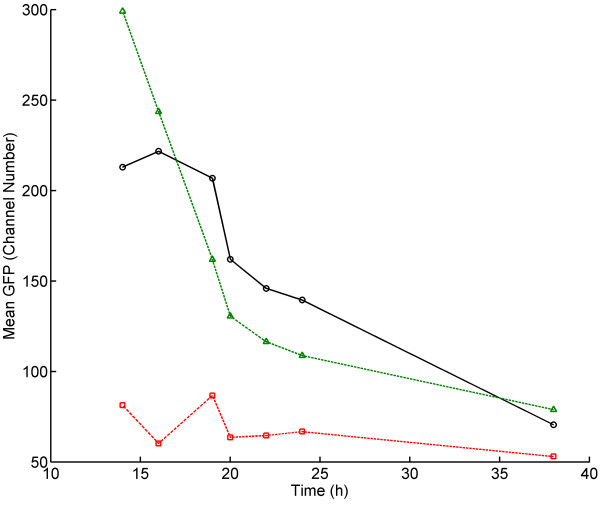
**Effect of freeze-thaw stress on GFP signal.** Mean GFP level of the entire population prior to freeze-thaw stress (*circles*) and mean GFP level of PI negative cells (*triangles*) and PI positive cells (*squares*) after freeze-thaw stress plotted against point of harvest during aerobic batch cultivation. The GFP signal was significantly decreased in cells that have been permeabilised by the stress exposure, hence making PI staining redundant to estimate number of cells with intact membranes.

Level of cell membrane robustness was also found to be correlated to cellular GFP level after exposure to freeze-thaw stress. Permeabilised cells significantly lost GFP fluorescence, while the PI negative subpopulation with intact cell membrane kept similar fluorescence intensity to untreated cells (Figure 5). The loss in GFP fluorescence in cells with permeabilised cell membrane may be due to leakage of intracellular GFP [31] and it may additionally be due to a decrease in intracellular pH which quenches the fluorescence signal [32]. Regardless of the specific cause, the measurable loss of GFP signal means that PI staining becomes redundant and that cell membrane robustness can be estimated by GFP level alone. In fact, this demonstrates that the reporter strain can be used as a dual reporter system for mapping heterogeneities in both growth (GFP level prior to stress exposure) and cell membrane robustness (distribution of subpopulations with high/low GFP level after stress exposure). The redundancy of PI was clear for the experimental set-up used in this study, and it may be useful for other investigations of cell membrane robustness on a single cell level; however, further experiments are needed to confirm that the dual nature of the reporter system also is applicable for other cases of physical stress. 

### Changes in cell membrane robustness by glucose perturbation

**Figure 6:**
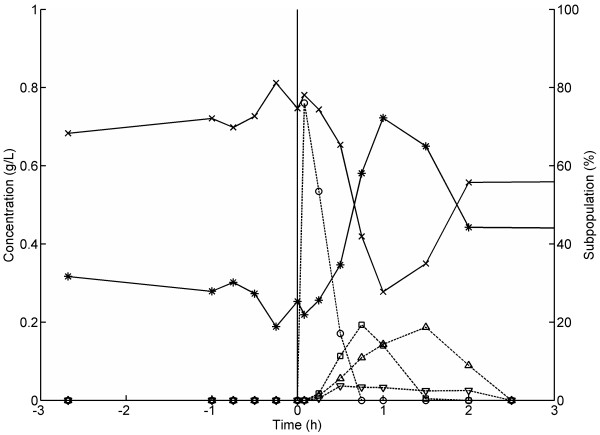
**Dynamic responses of subpopulations and metabolites in glucose gradient experiments mimicking large-scale cultivation conditions.** An aerobic glucose-limited chemostat was perturbed with a low concentration glucose pulse. Cells were sampled from the bioreactor and exposed to freeze-thaw stress and subsequently analysed with flow cytometry. Symbols: Subpopulation P1 (high GFP level, intact cell membranes) (*crosses*); Subpopulation P2 (low GFP level, permeabilised cell membranes) (*snowflakes*); Glucose (*open circles*); Acetate (*open diamonds*); Ethanol (*open squares*); Glycerol (*open triangles*).

**Figure 7:**
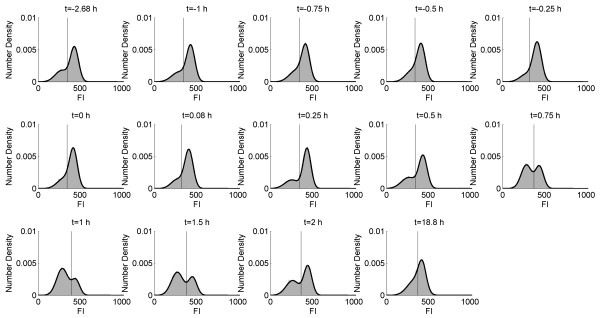
**GFP histograms illustrating the distribution of subpopulations with different degree of cell membrane robustness following the glucose pulse.** GFP histograms (*grey area*). FI = Fluorescence intensity. The black lines correspond to the fitted mixture of two Gaussian distributions. The vertical line corresponds to the subpopulation classification threshold.

To study how heterogeneity in cell membrane robustness of the population is influenced by a dynamic environment often seen in larger scale cultivations, an experiment mimicking glucose gradients was performed. Cells were grown in continuous mode with D = 0.2 h^-1^ (which is below the dilution rate where overflow metabolism occurs [33,34]) and subjected to a glucose pulse. Cells were harvested at different time points during the pulse and subsequently exposed to freeze-thaw stress. As demonstrated above, the GFP signal could directly be used as a measure of cell membrane robustness and no additional staining was applied for these experiments. During steady state, the mean fluorescence was constant and the cells displayed two separate subpopulations with varying degree of cell membrane robustness (ca 80-85% of subpopulation P1, high mean fluorescence, intact cell membrane; ca 15-20% of subpopulation P2, low mean fluorescence, permeabilised cell membrane) (Figures 6,7). The degree of cells with permeabilised cell membrane was thus significantly lower than for a population during batch growth on glucose, which is consistent with the inverse relationship between growth rate and cell robustness. Furthermore, the steady distribution of subpopulations demonstrates that continuous cultivation, where the environment is constant and the average cell population grows with a constant growth rate, is the preferred experimental setup when comparing physiology of different strains, since dynamic changes in physiology otherwise seen in batch mode are minimised. Continuous cultivation may also be used to investigate how subpopulation distribution is affected by growth rate as it allows for changing the growth rate while keeping other conditions constant and this knowledge can then be applied in development and design of a fed-batch process. 

At time point zero, a pulse of 10 mL highly concentrated glucose solution was added and the glucose concentration inside the bioreactor instantly increased from ca 0 g/L to 0.8 g/L. Directly after the pulse, glucose was consumed and ethanol, acetate and glycerol were produced at different rates, as has been observed previously [35]. After glucose depletion, ethanol and acetate consumption occurred. During the pulse, the cells rapidly experienced a variation from a low to a high glucose concentration, which is similar to what a cell may experience in fed-batch cultivations as the cell is transported from the bottom of a bioreactor – a low glucose environment – to the top, which normally is close to the feeding point and hence has a high glucose concentration [36]. Approximately 15 minutes after glucose was added, a drop in the P1 subpopulation (intact cell membranes) and an increase in the P2 subpopulation (permeabilised cell membranes) fractions were observed (Figures 6,7). The P2 subpopulation increased to 63% of the entire population 45 min after the glucose pulse was added. This demonstrates that a sudden change in glucose concentration has a profound effect on cell fitness distribution, since an increased number of cells with lower fluorescence and consequently lower cell membrane robustness emerged. The shift from steady state growth with D = 0.2 h^-1^ to batch mode and hence higher growth rate led to that a substantial part of cellular resources were redistributed to promote an increased growth rate, and consequently, the population became more sensitive to freeze-thaw stress. A difference in subpopulation distribution at steady states before and after the pulse was observed (68% P1 before compared to 59% P1 after the pulse), despite similar biomass and carbon dioxide profiles. The mechanism underlying the difference in subpopulation distribution at the different steady states before and after the pulse is not clear, however, it is probable that steady state after the pulse had not yet been reached on all levels despite constant biomass concentration, as has been observed previously [33].

## Conclusions

A dual reporter system in budding yeast was constructed and used to measure population heterogeneities in growth and cell membrane integrity after physical stress, using freeze-thaw stress as a model. A clear inverse correlation between growth and cell membrane robustness was observed and the two antagonistic phenotypes co-existed in the population, demonstrating that the population was prepared for different types of variations in environmental conditions.

Cells in stationary phase displayed the highest cell membrane robustness and tolerance to freeze-thaw stress. For cells that were actively proliferating, the percentage of the subpopulation with low cell membrane robustness was lower in continuous cultivation mode as compared to a population growing in batch mode. However, the low membrane robustness phenotype could be easily induced for cells growing under glucose limiting conditions by a sudden increase of glucose at a low concentration. Distribution of the two subpopulations with high and low membrane robustness was modulated quickly as a response to the low-level fluctuation in glucose concentration. This suggests that spatial heterogeneities of glucose concentration in a fermentation tank may be detrimental for cell membrane robustness and physical stress tolerance. Furthermore this demonstrates the importance of rapid methods for monitoring effects of small sudden changes on microbial cultures at a single-cell level

To the best of our knowledge this is the first time that the existence of subpopulations with different tolerance towards physical stress and a reporter system to analyse distributions of cell membrane robustness in budding yeast have been reported. The developed system is useful for optimisation studies for more efficient production in microbial cell factories or for optimising physical state of a population at point of harvest, thus increasing resistance to adverse conditions during downstream processing where intact cells are desired, for example for the production of baker’s yeast. Furthermore, a high fraction of cells with robust membrane phenotype may be important in secretory production of recombinant proteins where release of contaminating intracellular proteins is highly unwanted [37]. Contrary, in production processes having a cell-lysis step to liberate the product a high percentage of a less robust subpopulation may be preferred.

## Methods

### Strains

*Escherichia coli* strain MC1000 *araD139* Δ(*ara-leu*)*7679 galU galK lac 174 rpsL thi-1*[38] was used for subcloning before yeast transformation. Plasmids and *S. cerevisiae* strains are summarised in Table 1. All strains were stored in 15% glycerol stocks in liquid media at −80°C. *S. cerevisiae* strains were plated on YNB-agar plates (6.7 g/L yeast nitrogen base (Difco, USA), 20 g/L glucose and 20 g/L agar) and incubated for 2 days at 30°C before use. 

**Table 1:** ***S. cerevisiae*strains and plasmids used in this study**

**Strains**	**Relevant genotype**	**Ref.**
CEN.PK 113-5D	Mat**a** SUC2 MAL2-8^c^*ura3-52*	[39]
FE440	CEN.PK 113-5D with *ura3-52:*: URA3-P_RPL22A_-yEGFP-T_CYC1_ chromosomal integration	This study
FE522	CEN.PK 113-5D with *ura3-52:*:URA3 chromosomal integration	This study
**Plasmids**		
pBR322		[40,41]
pFe131	pBR322 with CEN1-ARS4-URA3	This study
pFe134	pFe131 with P_RPL22A_-yEGFP-T_CYC1_	This study

### Molecular biology techniques

PCR was performed using Phusion® DNA polymerase from Finnzymes (Espoo, Finland) and all other enzymes for cloning were purchased from Fermentas International Inc (Canada) and used following the recommendations of the manufacturer. Purification of DNA fragments from agarose gels was performed using the DNA Extraction Kit from Fermentas International Inc (Canada). Plasmid DNA was isolated from *E. coli* by using ZyppyTM Plasmid Miniprep Kit, Zymo Research (California, USA). Chromosomal DNA from *S. cerevisiae* was purified using YeaStarTM Genomic DNA Kit from Zymo Research (California, USA). Sequencing of DNA constructs was done by Macrogen (Seoul, Korea). Cells of *E. coli* were transformed by electroporation using a Bio-Rad Micropulser^TM^ and the recommended procedure of the manufacturer. *S. cerevisiae* cells were made competent, frozen in sorbitol buffer and transformed by electroporation according to the protocol of [Suga *et al.* 42]. Southern blotting was done with the digoxigenin method by Roche (Indianapolis, IN, USA) using a digoxigenin labeled yEGFP PCR probe and hybridization at 65°C.

### Construction of reporter strains

A 2200 bp EcoRI-PaeI fragment of CEN4-ARS1 was obtained by PCR with pCM188 as template [43] and a 1143 bp URA3 fragment flanked by *Pae*I and *Bam*HI/*Sal*I was obtained with pEMBLyex4 as template [44]. pBR322 [41] was cut with *Eco*RI and *Sal*I and the larger 4085 bp fragment was purified. The CEN4-ARS1 and URA3 fragments were cut with *Pae*I, ligated in vitro and cloned in the purified *Eco*RI-*Sal*I fragment of pBR322 resulting in plasmid pFe131. Then the RPL22A promoter was amplified as a 444 bp *Bam*HI-*Hind*III fragment with CEN.PK 113-5D chromosomal DNA as template, a 730 bp yEGFP3 fragment flanked by *Hind*III in the 5’ end and *Xho*I-*Xba*I sites divided by two stop codons in the 3’ end was amplified with pYGFP3 [17] as template and the CYC1 terminator was amplified as a 268 bp *Xba*I-*Sal*I fragment with pCM188 as template. The RPL22A promoter fragment was cut with *Hind*III, the yEGFP3 fragment was cut with *Hind*III and *Xba*I, the CYC1 terminator fragment was cut with *Xba*I and the three fragments were ligated in vitro and the combined 1442 bp fragment was purified from an agarose gel. This fragment was then cloned with *Bam*HI-SalI in pFe131 resulting in pFe134. For chromosomal integrations a 2585 bp URA3-RPL22A-yEGFP3-TCYC1 fragment was amplified with primers FP212 and FP169 with pFe134 as template and transformed to CEN.PK 113-5D resulting in strain FE440, whereas the control strain FE522 was obtained by transforming CEN.PK 113-5D with a 1143 bp URA3 fragment obtained by PCR with primers FP212 and FP213 and with pFe131 as template. Primers for chromosomal integrations are shown in Table 2. Correct chromosomal integration was verified by sequencing and Southern blotting. 

**Table 2:** **Primers used for*S. cerevisiae*chromosomal integrations**

**Primer**	**Sequence 5'-3'**
FP169, r	TATAAAGGCCATGAAGCTTTTTCTTTCCAATTT TTTTTTTTTCGTCATTA
	TAGAAATCATTACGACCGAGATTCCCGGGTAATTGGCCGCAAATTAAAGC
FP212, f	GATTCGGTAATCTCCGAGCAGAAGGAAGAACGA AGGAAGGAGCAC
	CAGCTATGACCATGATTACG
FP213, r	TTTTTCGTCATTATAGAAATCATTACGACCGAG ATTCCCGGGTAA
	TTTTTGATCGGGTAATAACTG

The underlined sequence is complementary to the PCR template; the remaining part of the sequence is complementary to the ura3-52 chromosomal site in CEN.PK113-5D.

### Batch cultivations

Batch cultivations were performed in duplicate using Sartorius 1 L bioreactors (Sartorius Stedim Biotech, Germany) with a working volume of 1.0 L. The pH and DOT electrode (Mettler Toledo, OH, USA) were calibrated according to standard procedures provided by the manufacturer. Inocula of *S. cerevisiae* strains were prepared by transferring colonies from fresh YNB-plates into 500 mL Erlenmeyer flasks containing 100 mL defined mineral media [45] supplemented with 10 g/L glucose and incubating in a shake incubator set to 150 rpm and 30°C. Precultures were grown until reaching mid exponential phase (approximately 10 hours) and then used directly for inoculation (starting OD_600nm_ = 0.001) of the bioreactor containing defined mineral media [45] supplemented with 5 g/L glucose. Cultivation conditions were set to the following; aeration 1 v/v/min; temperature 30°C; stirring 600 rpm and pH 5.0 (pH was controlled by automatic addition of 2 M KOH). Samples for OD_600nm_, high performance liquid chromatography (HPLC) and flow cytometry analysis were withdrawn approximately every 1 hour. Samples for OD_600_ were analysed directly while samples for HPLC were kept at −20°C. Samples for flow cytometry were centrifuged for 1 minute at 3000 *g* and 4°C, and resuspended in saline solution. Cells were then kept in saline solution on ice for maximum 45 minutes until they were stained with PI (10 μg/mL) [46]. In brief, cell samples were stained by the addition of PI stock solution and subsequently incubated in darkness for 20 min at room temperature and analysed by flow cytometry. As positive control for cells with permeabilised plasma membranes was ethanol (70%) treated cells (100% of cells were PI positive; PI >100 ch. nr.).

### Glucose gradient simulation

Continuous cultivations were performed using a Sartorius 2 L bioreactor (Sartorius Stedim Biotech, Germany) with a working volume of 1.5 L. Cultivation parameters were set as described above. After an initial batch phase, the continuous operation mode was started when glucose was nearly depleted and the carbon dioxide production started to peak. The dilution rate (D) was set to 0.2 h^-1^, which was below the dilution rate where overflow metabolism occurs [33]. Steady state was observed after feeding ca 5 reactor volumes, and biomass and carbon dioxide were constant for at least 20 hours. When steady state was reached, 10 mL of concentrated glucose was swiftly added to the bioreactor by using a sterile syringe, which resulted in an increase in glucose concentration from ca 0 g/L to 0.8 g/L. Samples were taken before, during and after glucose addition and analysed by HPLC. Cell samples were exposed to freeze-thaw stress as described below and analysed by flow cytometry.

### Freeze-thaw stress experiments

Samples were withdrawn from the bioreactor using a sterile syringe and then instantly mixed with an equal volume of 30% glycerol solution, resulting in a cell suspension with 15% glycerol. Cell suspensions were subjected to freezing by placement in a freezer set to −80°C. After complete freezing for at least 4 hours, cell samples were taken out of the freezer and placed in a water bath with controlled temperature at 37°C until samples were entirely thawed. After thawing, cells were centrifuged for 1 minute at 3000 *g* and 4°C, and resuspended in saline solution, and kept on ice for maximum 45 min until analysis. Cells were stained with PI (10 μg/mL) as described above and analysed by flow cytometry.

### Analyses

Growth was monitored by measuring OD_600nm_ with a Shimadzu UV mini 1240 spectrophotometer (Shimidzu, Kyoto, Japan). The concentrations of glucose, acetate, ethanol, glycerol, pyruvate and succinate were determined by HPLC (Agilent 1100, Agilent Technologies, CA, USA) with a 300 mm × 7.8 mm Aminex HPX-87 H ion exchange column (Bio-Rad, Hercules, CA, USA), refractive index detector (RID Agilent 1200, Agilent Technologies, CA, USA) and UV detector (Agilent 1100, Agilent Technologies, CA, USA) set to 210 nm. The mobile phase was 5 mM H_2_SO_4_ (aq.), temperature 60°C and flow rate 0.6 mL/min. The composition of the outgoing gas from batch cultivations was monitored by a 1311 Fast response Triple-gas monitor (Innova Air tech technologies, Ballerup, Denmark).

A BD FACSAria III (Becton-Dickinson, NJ, USA) flow cytometer was used for single-cell analysis. Excitation wavelength for the laser used was 488 nm. Fluorescence emission levels were measured using a band pass filter at 530/30 nm (FITC) and 616/23 (PI). Light scattering and fluorescence levels were standardized using 2.5 μm fluorescent polystyrene beads. 10,000 events were recorded with a rate of approximately 1,000 events per second. Processing and analysis of flow cytometry raw data was performed by using MatLab ® R2009b (The MathWorks, Inc., Natick, MA, USA). The measurement files, exported as fcs files by the flow cytomer FACSAria III, were imported into MatLab®, using a “fcs data reader” routine (by L.Balkay, University of Debrecen, Hungary), available on MatLab® File Exchange website. The classification of cells into a high and a low fluorescence subpopulation was based on fitting a gaussian mixture of two components to the GFP fluorescence histograms (using a nonlinear least square curve fitting algorithm available in MatLab® R2009b). The relative weight of the two gaussian distributions in the mixture were used as prior probabilities in the definition of the classification rule that minimizes the expected cost of misclassification [47].

## Competing interests

The authors declare that they have no competing interests.

## Authors’ contributions

MC and RLF participated in the design of the study, physiological experiments, flow cytometry analysis and drafted the manuscript. SH carried out the strain constructions. ALH participated in the physiological experiments and flow cytometry analysis. LL participated in the flow cytometry analysis. SJS participated in the design of the study. KVG participated in the design of the study and its coordination. AEL conceived the study, and participated in its design and coordination and helped to draft the manuscript. All authors read and approved the final manuscript.

## Supplementary Material

Additional file 1 **S1.** Percentile analysis. Click here for file

## References

[B1] Lencastre Fernandes R Nierychlo M Lundin L Pedersen AE Puentes Tellez PE Dutta A Carlquist M Bolic A Schapper D Brunetti AC Helmark S Heins AL Jensen AD Nopens I Rottwitt K Szita N Van Elsas JD Nielsen PH Martinussen J Sørensen SJ Lantz AE Gernaey KV Experimental methods and modeling techniques for description of cell population heterogeneity Biotechnol Adv 2011 29 575 599 10.1016/j.biotechadv.2011.03.007 21540103

[B2] Avery SV Microbial cell individuality and the underlying sources of heterogeneity Nat Rev Microbiol 2006 4 577 587 10.1038/nrmicro1460 16845428

[B3] Muller S Harms H Bley T Origin and analysis of microbial population heterogeneity in bioprocesses Curr Opin Biotechnol 2010 21 100 113 10.1016/j.copbio.2010.01.002 20138500

[B4] Enfors SO Jahic M Rozkov A Xu B Hecker M Jurgen B Kruger E Schweder T Hamer G O'Beirne D Noisommit-Rizzi N Reuss M Boone L Hewitt C McFarlane C Nienow A Kovacs T Trägårdh C Fuchs L Revstedt J Friberg PC Hjertager B Blomsten G Skogman H Hjort S Hoeks F Lin HY Neubauer P Van der Lans R Luyben K Physiological responses to mixing in large scale bioreactors J Biotechnol 2001 85 175 185 10.1016/S0168-1656(00)00365-5 11165362

[B5] Hewitt CJ Nebe-Von Caron G Axelsson B McFarlane CM Nienow AW Studies related to the scale-up of high-cell-density E. coli fed-batch fermentations using multiparameter flow cytometry: effect of a changing microenvironment with respect to glucose and dissolved oxygen concentration Biotechnol Bioeng 2000 70 381 390 10.1002/1097-0290(20001120)70:4<381::AID-BIT3>3.0.CO;2-0 11005920

[B6] Díaz M Herrero M García LA Quirós C Application of flow cytometry to industrial microbial bioprocesses Biochem Eng J 2010 48 385 407 10.1016/j.bej.2009.07.013

[B7] Mattanovich D Borth N Applications of cell sorting in biotechnology Microb Cell Fact 2006 5 12 10.1186/1475-2859-5-12 16551353 PMC1435767

[B8] Lara AR Galindo E Ramirez OT Palomares LA Living with heterogeneities in bioreactors: understanding the effects of environmental gradients on cells Mol Biotechnol 2006 34 355 381 10.1385/MB:34:3:355 17284782

[B9] Delvigne F Boxus M Ingels S Thonart P Bioreactor mixing efficiency modulates the activity of a prpoS::GFP reporter gene in E. coli Microb Cell Fact 2009 8 15 10.1186/1475-2859-8-15 19243588 PMC2650683

[B10] Bylund F Collet E Enfors SO Larsson G Substrate gradient formation in the large-scale bioreactor lowers cell yield and increases by-product formation Bioproc Eng 1998 18 171 180 10.1007/s004490050427

[B11] Lewis JG Learmonth RP Watson K Role of growth phase and ethanol in freeze-thaw stress resistance of Saccharomyces cerevisiae Appl Environ Microbiol 1993 59 1065 1071 8476282 10.1128/aem.59.4.1065-1071.1993 PMC202239

[B12] Klis FM Boorsma A De Groot PW Cell wall construction in Saccharomyces cerevisiae Yeast 2006 23 185 202 10.1002/yea.1349 16498706

[B13] Gasch AP Hohmann S, Mager WH The environmental stress response: a common yeast response to environmental stresses Yeast Stress Responses 2002 Springer, Heidelberg 11 70 Hohmann S. (Series Editor)

[B14] Zakrzewska A van Eikenhorst G Burggraaff JE Vis DJ Hoefsloot H Delneri D Oliver SG Brul S Smits GJ Genome-wide analysis of yeast stress survival and tolerance acquisition to analyze the central trade-off between growth rate and cellular robustness Mol Biol Cell 2011 22 4435 4446 10.1091/mbc.E10-08-0721 21965291 PMC3216668

[B15] Lidstrom ME Konopka MC The role of physiological heterogeneity in microbial population behavior Nat Chem Biol 2010 6 705 712 10.1038/nchembio.436 20852608

[B16] Ferrer-Miralles N Domingo-Espin J Corchero JL Vazquez E Villaverde A Microbial factories for recombinant pharmaceuticals Microb Cell Fact 2009 8 17 10.1186/1475-2859-8-17 19317892 PMC2669800

[B17] Cormack BP Bertram G Egerton M Gow NA Falkow S Brown AJ Yeast-enhanced green fluorescent protein (yEGFP)a reporter of gene expression in Candida albicans Microbiology 1997 143 303 311 10.1099/00221287-143-2-303 9043107

[B18] Day RN Davidson MW The fluorescent protein palette: tools for cellular imaging Chem Soc Rev 2009 38 2887 2921 10.1039/b901966a 19771335 PMC2910338

[B19] Regenberg B Grotkjaer T Winther O Fausboll A Akesson M Bro C Hansen LK Brunak S Nielsen J Growth-rate regulated genes have profound impact on interpretation of transcriptome profiling in Saccharomyces cerevisiae Genome Biol 2006 7 R107 10.1186/gb-2006-7-11-r107 17105650 PMC1794586

[B20] Fazio A Jewett MC Daran-Lapujade P Mustacchi R Usaite R Pronk JT Workman CT Nielsen J Transcription factor control of growth rate dependent genes in Saccharomyces cerevisiae: a three factor design BMC Genomics 2008 9 341 10.1186/1471-2164-9-341 18638364 PMC2500033

[B21] Brauer MJ Huttenhower C Airoldi EM Rosenstein R Matese JC Gresham D Boer VM Troyanskaya OG Botstein D Coordination of growth rate, cell cycle, stress response, and metabolic activity in yeast Mol Biol Cell 2008 19 352 367 10.1091/mbc.E07-08-0779 17959824 PMC2174172

[B22] Warner JR Vilardell J Sohn JH Economics of ribosome biosynthesis Cold Spring Harb Symp Quant Biol 2001 66 567 574 10.1101/sqb.2001.66.567 12762058

[B23] Mateus C Avery SV Destabilized green fluorescent protein for monitoring dynamic changes in yeast gene expression with flow cytometry Yeast 2000 16 1313 1323 10.1002/1097-0061(200010)16:14<1313::AID-YEA626>3.0.CO;2-O 11015728

[B24] Roostalu J Joers A Luidalepp H Kaldalu N Tenson T Cell division in Escherichia coli cultures monitored at single cell resolution BMC Microbiol 2008 8 68 10.1186/1471-2180-8-68 18430255 PMC2377270

[B25] Slavov N Botstein D Coupling among growth rate response, metabolic cycle, and cell division cycle in yeast Mol Biol Cell 2011 22 1997 2009 10.1091/mbc.E11-02-0132 21525243 PMC3113766

[B26] Growth rate response, http://genomics-pubs.princeton.edu/grr/

[B27] Sumner ER Avery AM Houghton JE Robins RA Avery SV Cell cycle- and age-dependent activation of Sod1p drives the formation of stress resistant cell subpopulations within clonal yeast cultures Mol Microbiol 2003 50 857 870 10.1046/j.1365-2958.2003.03715.x 14617147

[B28] Brauer MJ Saldanha AJ Dolinski K Botstein D Homeostatic adjustment and metabolic remodeling in glucose-limited yeast cultures Mol Biol Cell 2005 16 2503 2517 10.1091/mbc.E04-11-0968 15758028 PMC1087253

[B29] Cipollina C Alberghina L Porro D Vai M SFP1 is involved in cell size modulation in respiro-fermentative growth conditions Yeast 2005 22 385 399 10.1002/yea.1218 15806610

[B30] Ando A Nakamura T Murata Y Takagi H Shima J Identification and classification of genes required for tolerance to freeze-thaw stress revealed by genome-wide screening of Saccharomyces cerevisiae deletion strains FEMS Yeast Res 2007 7 244 253 10.1111/j.1567-1364.2006.00162.x 16989656

[B31] Delvigne F Brognaux A Francis F Twizere JC Gorret N Sorensen SJ Thonart P Green fluorescent protein (GFP) leakage from microbial biosensors provides useful information for the evaluation of the scale-down effect Biotechnol J 2011 6 968 978 10.1002/biot.201000410 21695786

[B32] Kneen M Farinas J Li Y Verkman AS Green fluorescent protein as a noninvasive intracellular pH indicator Biophys J 1998 74 1591 1599 10.1016/S0006-3495(98)77870-1 9512054 PMC1299504

[B33] Van Hoek P Van Dijken JP Pronk JT Effect of specific growth rate on fermentative capacity of baker's yeast Appl Environ Microbiol 1998 64 4226 4233 9797269 10.1128/aem.64.11.4226-4233.1998 PMC106631

[B34] Carlsen M Jochumsen KV Emborg C Nielsen J Modeling the growth and proteinase A production in continuous cultures of recombinant Saccharomyces cerevisiae Biotechnol Bioeng 1997 55 447 454 10.1002/(SICI)1097-0290(19970720)55:2<447::AID-BIT22>3.0.CO;2-C 18636503

[B35] Flikweert MT Kuyper M van Maris AJ Kotter P van Dijken JP Pronk JT Steady-state and transient-state analysis of growth and metabolite production in a Saccharomyces cerevisiae strain with reduced pyruvate-decarboxylase activity Biotechnol Bioeng 1999 66 42 50 10.1002/(SICI)1097-0290(1999)66:1<42::AID-BIT4>3.0.CO;2-L 10556793

[B36] George S Larsson G Olsson K Enfors SO Comparison of the baker's yeast process performance in laboratory and production scale Bioproc Eng 1998 18 135 142 10.1007/PL00008979

[B37] Porro D Sauer M Branduardi P Mattanovich D Recombinant protein production in yeasts Mol Biotechnol 2005 31 245 259 10.1385/MB:31:3:245 16230775

[B38] Casabadan MJaC SN Analysis of gene control signals by DNA fusion and cloning in Escherichia coli J Mol Biol 1980 138 179 207 10.1016/0022-2836(80)90283-1 6997493

[B39] van Dijken JP Bauer J Brambilla L Duboc P Francois JM Gancedo C Giuseppin ML Heijnen JJ Hoare M Lange HC Madden EA Niederberger P Nielsen J Parrou JL Petit L Porro D Reuss M Van Riel N Rizzi M Steensma HY Verrips CT Vindelov J Pronk JT An interlaboratory comparison of physiological and genetic properties of four Saccharomyces cerevisiae strains Enzyme Microb Technol 2000 26 706 714 10.1016/S0141-0229(00)00162-9 10862876

[B40] Watson N A new revision of the sequence of plasmid pBR322 Gene 1988 70 399 403 10.1016/0378-1119(88)90212-0 3063608

[B41] Bolivar F Rodriguez RL Greene PJ Betlach MC Heyneker HL Boyer HW Crosa JH Falkow S Construction and characterization of new cloning vehicles. II. A multipurpose cloning system Gene 1977 2 95 113 10.1016/0378-1119(77)90000-2 344137

[B42] Suga M Isobe M Hatakeyama T Cryopreservation of competent intact yeast cells for efficient electroporation Yeast 2000 16 889 896 10.1002/1097-0061(200007)16:10<889::AID-YEA582>3.0.CO;2-R 10870100

[B43] Gari E Piedrafita L Aldea M Herrero E A set of vectors with a tetracycline-regulatable promoter system for modulated gene expression in Saccharomyces cerevisiae Yeast 1997 13 837 848 10.1002/(SICI)1097-0061(199707)13:9<837::AID-YEA145>3.0.CO;2-T 9234672

[B44] Murray JA Scarpa M Rossi N Cesareni G Antagonistic controls regulate copy number of the yeast 2 mu plasmid EMBO J 1987 6 4205 4212 2832156 10.1002/j.1460-2075.1987.tb02768.x PMC553905

[B45] Verduyn C Postma E Scheffers WA Van Dijken JP Effect of benzoic acid on metabolic fluxes in yeasts: a continuous-culture study on the regulation of respiration and alcoholic fermentation Yeast 1992 8 501 517 10.1002/yea.320080703 1523884

[B46] Kacmar J Zamamiri A Carlson R Abu-Absi NR Srienc F Single-cell variability in growing Saccharomyces cerevisiae cell populations measured with automated flow cytometry J Biotechnol 2004 109 239 254 10.1016/j.jbiotec.2004.01.003 15066762

[B47] Johnson RA Richard DW Applied multivariate statistical analysis 2007 Pearson International Edition, Upper Saddle River

