# Supplementary material for: Physiological heterogeneities in microbial populations and implications for physical stress tolerance
Source: Microb Cell Fact. 2012 Jul 16;11:94. doi: 10.1186/1475-2859-11-94 (PMC3443036; doi:10.1186/1475-2859-11-94)

SUPPLEMENTARY MATERIAL

S1 – Percentile Analysis

The percentile analysis performed in this work is based on separating cells (events in the flow cytometer) according to their forward scatter signal (FSC).

Ten FSC intervals containing 10% of the total 10000 events are define by the 10th up to 100th percentiles as illustrated in Figure S1. The mean FSC as well as mean fluorescence intensity (FI), corresponding to the GFP content of the cells, is estimated by averaging the signals measured for each event belonging to a given FSC percentile interval. Also the distributions of GFP within each percentile interval can be analysed by plotting the corresponding histogram (Figure S2)

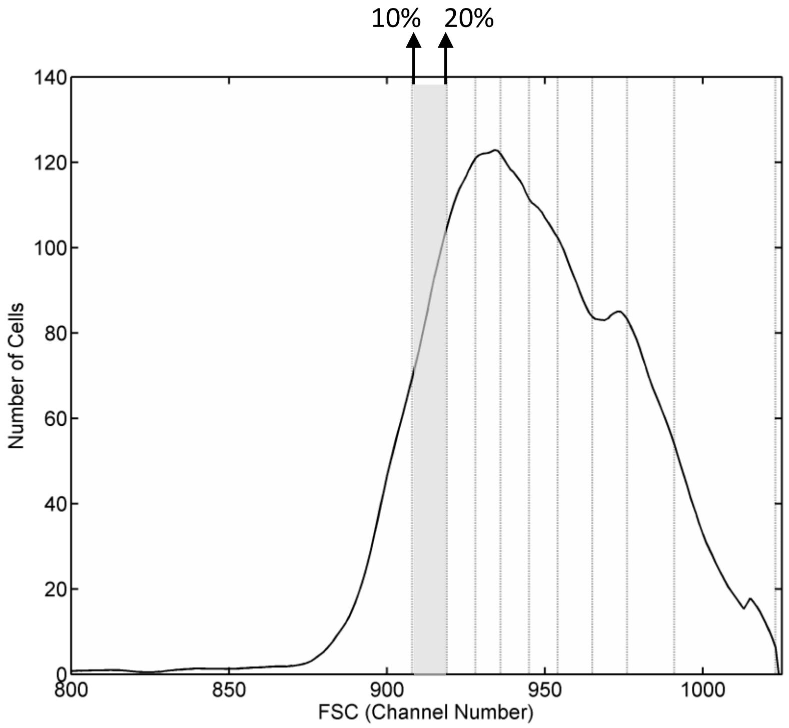

Figure S1 - Percentile analysis. A representative FSC histogram obtained from flow cytometry analysis of a cell population that was sampled during aerobic batch cultivation. The FSC histogram was divided in 10%-percentiles as indicated by the vertical lines. The mean fluorescence intensity (GFP content) for example for the percentile interval 10-20 % was calculated by averring the FI for the cells presenting a FSC between these percentiles, i.e within the gray box.

S2. Distribution of GFP for each FSC percentile interval. GFP histograms of gated cells based on the FSC 10%-percentiles as illustrated in Figure S1.

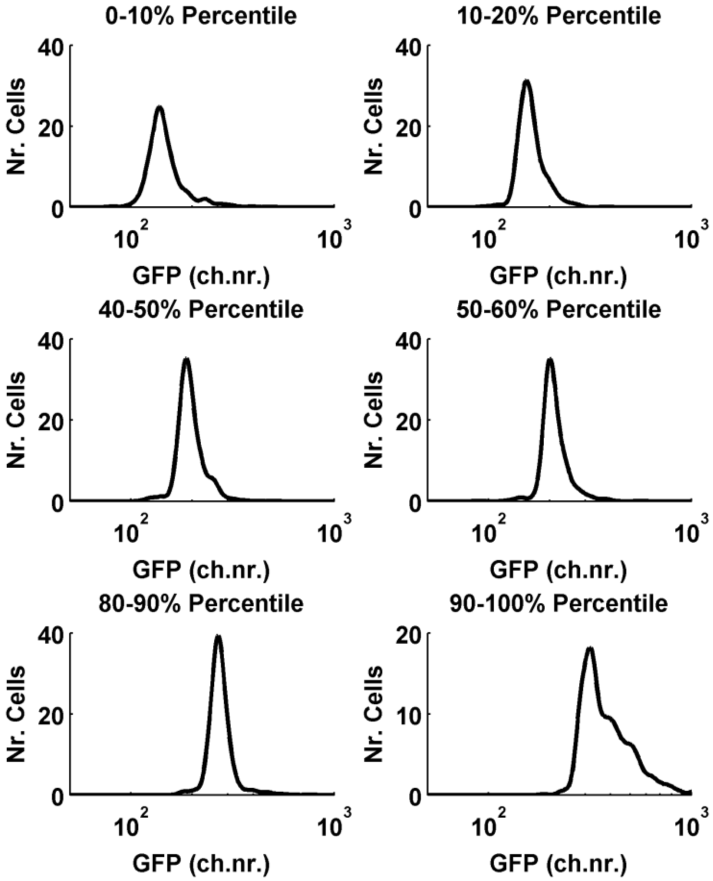

Supplement: Additional file 1 — S1. Percentile analysis. [file 1475-2859-11-94-S1.pdf]
